# Dr. David H. Reed

**Published:** 1903-03

**Authors:** 


Dr. David H. Reed, U. of B. '83, of Remsen, N. Y., died at his home January 9, 1903, after a long illness, aged 48 years.

